# Plant Virus Ecology

**DOI:** 10.1371/journal.ppat.1003304

**Published:** 2013-05-23

**Authors:** Marilyn J. Roossinck

**Affiliations:** 1 Departments of Plant Pathology and Environmental Microbiology, and Biology, Pennsylvania State University, University Park, Pennsylvania, United States of America; 2 Sir Walter Murdoch, Murdoch University, Perth, Western Australia; University of Florida, United States of America

Viruses have generally been studied either as disease-causing infectious agents that have a negative impact on the host (most eukaryote-infecting viruses), or as tools for molecular biology (especially bacteria-infecting viruses, or phage). Virus ecology looks at the more complex issues of virus-host-environment interactions. For plant viruses this includes studies of plant virus biodiversity, including viruses sampled directly from plants and from a variety of other environments; how plant viruses impact species invasion; interactions between plants, viruses and insects; the large number of persistent viruses in plants that may have epigenetic effects; and viruses that provide a clear benefit to their plant hosts (mutualists). Plants in a non-agricultural setting interact with many other living entities such as animals, insects, and other plants, as well as their physical environment. Wild plants are almost always colonized by a number of microbes, including fungi, bacteria and viruses. Viruses may impact any of these interactions [1].

## 1. Plant Virus Biodiveristy

About 15 years ago I heard a prominent plant virologist declare that surely we had discovered most, if not all of the plant viruses in the world by now! This was based on over 100 years of studying the disease-causing viruses of crop plants. The first virus ever studied, *Tobacco mosaic virus*, was found in a search for the causal agent of a mosaic disease in tobacco [2]. However, the study of viruses in non-crop hosts is only now being done, and the results are showing that contrary to the learned opinion of some scientists, we know very little about the viruses in plants [3]. Many viral studies have used a metagenomic approach for studying biodiversity, where environmental samples are analyzed for virus sequences after some enrichment for viruses, but plant virologists have used a different approach, where individual plants have been sampled [4]. This allows further ecological studies to be done.

The most recent report of the International Committee for the Taxonomy of Viruses, a group that approves scientific names for viruses, lists only about 900 species of plant viruses [5]. Preliminary studies from wild plants indicate that thousands of new viruses are yet to be discovered in plants [3], [4]. Persistent plant viruses, which have been poorly studied and have very few known phenotypes (see point 5), make up about half of all viruses found in wild plants. In addition, while relatives of known acute plant viruses are found, they generally do not cause any apparent disease in wild plants, even though they may cause disease in experimental plants [6], or nearby crop plants.

A number of metagenomic studies have identified plant-like viruses in a wide variety of samples, including feces of humans and other mammals; fresh water, sea water and reclaimed water; soil from rice paddies; and plant-feeding insects ([3], and references therein). Many of the viruses from these environments are known plant viruses, and probably entered the environment through animal ingestion of plants and subsequent passing of the viruses through the gut. All of the viruses found in these places are from genera with very stable capsid structures, such as *Tobamoviruses*. A prevalent virus in waste water is *Pepper mild mottle virus* [7], a virus also found in human feces, and reported to be associated with intestinal distress in humans [8], although, since this virus is also found in most preparations of hot peppers, it seems more likely that the intestinal distress is caused by the ingestion of hot peppers. The prevalence of *Pepper mild mottle virus* in human feces, and its stability in the environment, led to a proposal to use it as an easily assayed indicator of groundwater contamination [7].

## 2. Plant Viruses and Invasive Species

In general invasive species can be more robust in a new environment because they have left behind pathogens in their native habitat, a phenomenon known as pathogen release [9]. Invasive species of plants can be aided in their invasions by plant viruses in a number of different ways. Invasives may carry viruses in inapparent infections that cause disease in their native competitors, or they may be more tolerant than natives of viruses that are already in the environment [10]. Other mechanisms include a role of invasive plants in the ecology of plant virus vectors. One study showed that an invasive grass species resulted in increased populations of the aphid vector for plant viruses that was detrimental to a native grass species [11]. Another alternative, not described in the literature but quite plausible, is that invasive species carry beneficial viruses that give them a competitive edge in comparison to native species.

## 3. Viruses, Plants and Insects

Since plants are not generally mobile, their viruses must be transmitted by vectors. These can include non-specific mechanical vectors such as lawnmowers or pruning tools, or the teeth of grazing animals, but a majority of acute plant viruses are vectored by plant-feeding insects. Viruses can make plants more attractive to insects. Recent studies have shown that virus infection can affect the volatile compounds the plants produce, and this in turn can attract insects. In some cases virus-infected plants are better hosts for insects, resulting in increased feeding, whereas in other cases virus-infected plants are poor hosts, and insects leave quickly after probing the plants [12]. Although few studies have been done, there seems to be an interesting correlation between the type of transmission and the quality of the host for insect feeding. Viruses that are transmitted in a non-persistent manner (i.e., the insect acquires and transmits the virus rapidly through simple probing) seem to make the plants poorer hosts, whereas viruses that are transmitted in a persistent manner (i.e., the insect is viruliferous for a long period of time and the virus is usually processed through the gut of the insect) become better quality hosts for insects [12]. This makes sense from the virus-centric viewpoint: if the transmission is rapid it is better for the insect to quickly move off to a new plant, whereas if the transmission requires a stronger insect association increased feeding will increase the chances of proper acquisition [12].

In another recent report plant viruses were found to modify insect behavior more directly. Aphids that had already acquired *Barley yellow dwarf virus* were attracted to uninfected plants, whereas aphids that had not acquired the virus were attracted to infected plants [13], another striking behavior modification that can enhance the spread of the virus.

## 4. Persistent Plant Viruses

While most of the well-studied viruses in crop plants are acute (i.e., they induce an acute infection that is resolved over time, usually through host death or recovery), a majority of viruses in wild plants appear to have a persistent lifestyle [14]. They remain with their plant hosts indefinitely and are transmitted vertically at very high rates, approaching 100%. Persistent viruses are also found in crops, including beans, peppers and rice, where they have been studied more extensively [14]. They occur in virus families that have other members that infect fungi, including fungi that colonize plants. In most cases no function has been attributed to these viruses in plants, but one partitivirus, *White clover cryptic virus 2*, can help regulate nodulation in the presence of nitrogen. The coat protein of the virus has the same effect when expressed in a different legume, lotus [15]. It is likely that other viruses also contribute to their hosts as cytoplasmic epigenetic elements, but this has not been studied much [14]. Sequences of some persistent viruses are found in the genomes of several different plants [16], [17]. Interestingly, these sequences have not been found in the genomes of plants with cytoplasmic versions of persistent viruses, although the study of persistent plant viruses is certainly not complete. It is possible that the genomic sequences are remnants of past persistent virus infections that have somehow been cleared. Alternatively, the integrated sequences could provide protection against infection by similar viruses. The origins of these viruses are unknown, but their similarity to fungal viruses implies transmission across kingdoms. Phylogenetic analyses of the *Partitiviridae* and *Endornaviridae* families of persistent plant and fungal viruses support the transmission of these viruses among plant and fungal hosts. Since many fungi colonize plants, this could occur during these interactions [14].

## 5. Mutualistic Viruses of Plants

**Figure 1:**
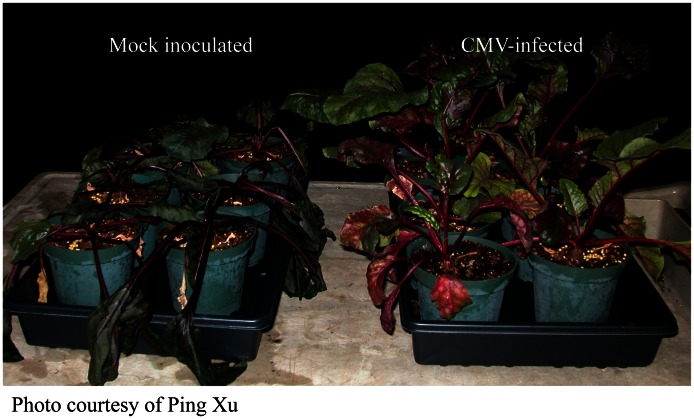
Virus infection confers cold tolerance to red beet plants. Plants on the left were inoculated with buffer only, plants are the right were infected with *Cucumber mosaic virus*. Plants were subjected to sub-freezing temperatures at night, to mimic conditions at the beginning or end of a growing season. Virus infected plants survived the cold treatment, whereas uninfected controls died.

The word “virus” usually conjures up images of rows of hospital beds during the flu epidemics, or barns full of dying chickens. For plants most people think of fields of yellow or dying crops. Recent studies suggest that most viruses may not cause obvious disease [3], and some are clearly beneficial. For plants, several acute viruses induce drought tolerance, an important trait in a changing environment, and *Cucumber mosaic virus* confers cold tolerance in red beets (Figure 1) [18]. Although the mechanisms for drought and cold tolerance are not clear, a number of potential osmoprotectants are increased in virus-infected plants [18]. How common are beneficial viruses in plants? We simply don't know, because this has not been studied. However, plants are capable of adaptation to extreme environments, such as in geothermal soils in Yellowstone National Park. Plants in this environment harbor fungal endophytes that are infected with a virus: all three partners are required for thermal tolerance [19]. Viruses, with their extreme diversity, are likely to be capable of contributing new genetic material to a system, and may be common factors in rapid adaptation. With our changing climate, making use of this potential role for viruses could become important for agriculture.
